# Cripto-1 expression in patients with clear cell renal cell carcinoma is associated with poor disease outcome

**DOI:** 10.1186/s13046-019-1386-6

**Published:** 2019-08-27

**Authors:** Yi-Jun Xue, Song-Ning Chen, Wei-Guang Chen, Geng-Qing Wu, Yun-Feng Liao, Jian-Bin Xu, Hao Tang, Shui-Hua Yang, Shui-Yong He, Yun-Fei Luo, Zhi-Hui Wu, Hai-Wen Huang

**Affiliations:** 1 Department of Urology, Central People’s Hospital of Zhanjiang, Guangdong Medical University, Zhanjiang, No.236, Yuanzhu Road, Zhanjiang, 524045 Guangdong Province People’s Republic of China; 2 Department of Urology, First Affiliated Hospital of Gannan Medical University, Ganzhou, 341000 People’s Republic of China

**Keywords:** Clear cell renal cell carcinoma, Cripto-1, Prognosis

## Abstract

**Background:**

Cripto-1 (CR-1) has been reported to be involved in the development of several human cancers. The potential role of CR-1 in clear cell renal cell carcinoma (ccRCC) is still not clear.

**Methods:**

CR-1 expression was evaluated in ccRCC tissues by Real-time quantitative PCR, Western blot and immunohistochemistry. Serum levels of CR-1 were tested by enzyme-linked immunosorbent assay (ELISA). The clinical significance of CR-1 was analyzed. The effects of CR-1 on cell proliferation, migration, invasion and angiogenesis were investigated in ccRCC cell lines in vitro and in vivo, and markers of the epithelial -mesenchymal transition (EMT) were analyzed. The impact of CR-1 on Wnt/β-catenin signaling pathway was also evaluated in vitro and in vivo.

**Results:**

CR-1 expression was elevated in ccRCC tumor tissues and serum samples. CR-1 expression was correlated with aggressive tumor phenotype and poor survival. Ectopic expression of CR-1 significantly promoted cell proliferation, migration, invasion and angiogenesis whereas knockdown of CR-1 inhibited these activities both in vitro and in vivo. Moreover, we found that CR-1 induced EMT and activated Wnt/β-catenin signaling pathway both in vitro and in vivo.

**Conclusions:**

These results suggest that CR-1 is likely to play important roles in ccRCC development and progression, and that CR-1 is a prognostic biomarker and a promising therapeutic target for ccRCC.

## Background

Renal cell carcinoma (RCC), the most deadly genitourinary cancer, accounts for about 3–4% of all adult malignant neoplasms worldwide [1]. Clear cell RCC (ccRCC) is the most prevalent histological variant, comprising 80–90% of all RCC cases [2]. While surgical intervention is effective for the majority of patients presenting with localized disease, about 30% of patients are initially diagnosed with metastatic diseases [3, 4]**.** In addition, up to 40% of RCC patients will develop metastatic disease after nephrectomy [5]. Commonly used clinicopathological parameters, such as TNM classification system and Fuhrman nuclear grade, provide robust prognostic value [6]. However, they can’t precisely predict a reliable result since similar TNM stage and Fuhrman grade may have considerable variability in survival [7]. The identification of RCC molecular markers that can improve early tumor detection and predict patient prognosis is warranted. Although long-standing efforts toward marker discovery and validation for RCC, there is still a lack of ideal markers that can be widely used in clinical practice.

Human Cripto-1 (CR-1), also called teratocarcinoma-derived growth factor-1 (TDGF-1) [8], is a member of the glycosylphosphatidylinositol (GPI)-anchored signaling protein family [9, 10], which acts as a coreceptor for the transforming growth factor beta (TGF-β) ligands, such as Nodal and growth differentiation factors 1 (GDF1) and 3 (GDF3). Structurally, CR-1 contain a signal sequence for extracellular secretion, a modified epidermal growth factor (EGF)-like domain, a conserved cysteine-rich domain (CFC-motif) and a short hydrophobic carboxy-terminus [11, 12]. CR-1 plays important roles during early embryonic evolution; however, is absent or minimally expressed in normal mature cells [13]. Previous research suggests that CR-1 is overexpressed in several types of human cancers such as gastric cancer [14], glioblastoma [15], bladder cancer [16], breast cancer [17], hepatocellular carcinoma [18], lung cancer [19, 20] and esophageal squamous cell carcinoma [21, 22]. More importantly, the atypical expression of CR-1 has been shown to be connected with clinically aggressive behaviour and patients’ survival in these cancers. Evidence also suggests that the level of CR-1 is elevated in the circulating serum of patients with lung cancer [23], hepatocellular carcinoma [24], breast and colon carcinomas [25]. Thus, it may serve as a promising biomarker for cancer diagnosis and prognosis. In addition, previous studies have illustrated that CR-1 plays an oncogenic function during carcinogenesis by boosting cell proliferation, survival, migration and invasion, as well as inducing epithelial-to- mesenchymal transition (EMT) and tumor angiogenesis [26]. Nevertheless the expression of CR-1 in ccRCC has not been elucidated and its clinical value in ccRCC is still indistinct.

Accordingly, the present study aimed to evaluate the clinical significance of CR-1 in both serum and tumor tissues in ccRCC. In addition, we investigated the biological role of CR-1 in cultured ccRCC cells. We found CR-1 expression was elevated in ccRCC cell lines, tumor tissues, and serum samples from ccRCC patients. Expression of CR-1 was related to clinicopathological features and prognosis in ccRCC patients. Downregulation of CR-1 by RNA inference significantly decreased proliferation, migration, invasion and angiogenesis of ccRCC cells in vitro and restrained tumorigenesis and metastasis in vivo. Conversely, ectopic expression of CR-1 in ccRCC cells noticeably enhanced these effects. Also, we found that CR-1 induced EMT and activated Wnt/β-catenin signaling pathway. These outcomes indicate that CR-1 plays a crucial role in ccRCC metastasis and progression and could be utilized as a latent prognostic biomarker of survival and a novel therapeutic target in patients with ccRCC.

## Materials and methods

### Patients and samples

Paraffin-embedded, archived ccRCC and matched adjacent non-tumor tissues used for immunohistochemistry (IHC) were acquired from 205 ccRCC patients undergoing nephrectomy at the Department of Urology, the 1st Affiliated Hospital of Gannan Medical University between 2005 and 2014. Furthermore, histologically normal samples of kidney tissue obtained from 8 patients with trauma nephrectomy were used as controls for IHC. Thirty-eight pairs of fresh ccRCC tissues and their corresponding adjacent non-tumor tissues used for real-time quantitative PCR (qRT-PCR) and Western blot analyses were collected during surgery at the same hospital between February 2017 and December 2017. After surgical removal, all the fresh tissue samples were promptly snap-frozen in liquid nitrogen and stored them at − 80 °C until ready for RNA or protein extraction. For the enzyme-linked immunosorbent assays (ELISA), serum samples were gathered from these patients before and after surgery. Postoperative serum samples were collected 4 weeks or more after surgery. Additionally, serum samples from 35 healthy individuals were used as a control for the ELISA assay. The patients were chosen based on the following criteria: 1) unilateral, sporadic, non-cystic, pathologically confirmed ccRCC; 2) no history of other malignancies; and 3) availability of detailed clinicopathologic data. Those who had the perioperative mortalities, preoperative anticancer treatment, and samples necrosis area > 80% were excluded from the investigation. All of the cases were staged in the light of the 2010 AJCC TNM classification and nuclear grade was assessed according to the Fuhrman criteria. This study was approved by the Ethics Committee of 1st Affiliated Hospital of Gannan Medical University (Ganzhou, China), and was performed in strict accordance with the approved guidelines and regulations. Written informed consent was achieved from all the patients. Patients were followed up routinely every 3 months during the first 2 years, every 6 months during the following 3 years, and then yearly thereafter. Overall survival (OS) was characterized as the interval between date of surgery and date of death. Recurrence-free survival (RFS) was characterized as the interval between date of surgery and date of recurrence. Totally 24 patients were ruled out in the RFS analysis owing to preoperational metastases. OS data were censored if patients were alive at the last follow-up date and RFS data were censored if recurrence was not observed during the follow-up period.

### qRT-PCR assay

qRT-PCR was carried out as previously described [27, 28].Total RNA was isolated with TRIzol reagent (Invitrogen, Carlsbad, CA, USA) according to the instructions of manufacturer. The isolated RNA was treated with RNase-free DNase I (Roche) for 15–30 min**.** First-strand cDNA was generated with the PrimeScript RT reagent kit (Takara, Dalian, China). qRT-PCR was performed by the ABI PRISM 7300 (Applied Biosystems, Foster City, CA, USA). The PCR primers used were as follows: CR-1 forward 5′-GATACAGCACAGTAAGGAGC-3′, reverse 5′-TAGTTCTGG AGTCCT GGAAG-3′, β-actin forward 5′-ACTGGAACGGTGAAGGTGAC-3′, reverse 5′-AGAGAAG TGGGG TGGCTTTT-3′. β-actin was utilized as a reference gene. Threshold cycle (Ct) values of the samples were determined, and the 2^-∆∆Ct^ method was employed for relative levels of CR-1 mRNA.

### Western blot assay

Western blot was carried out as previously described [29]. The primary antibodies utilized in this study included the following: anti-E-cadherin, anti-Vimentin, anti-ZEB-1, anti-MMP-9, anti-CyclinD1, anti-MMP-2, anti-Snail, anti-β-catenin, anti-p-GSK3β, anti-GSK3β, anti-C-myc (all 1:1000; Cell Signaling Technology, Beverly, MA), anti-CR-1 (1:500, Abcam, Cambridge, UK), anti-N-cadherin (1:500; Cell Signaling Technology), anti-VEGF (1:500; Santa Cruz Biotechnology, Santa Cruz, CA), anti-VEGF neutralizing antibody (R&D system). and anti-β-actin (1:2000; Santa Cruz Biotechnology).

### IHC assay

Tissue sections (4 μm thick) are cut from paraffin-embedded blocks. The primary antibody was rabbit polyclonal CR-1 antibody (1:200; Abcam, Cambridge, UK). IHC assay was done in accordance with a previously described method [30]. The IHC was evaluated in view of a combined score of the extent (%) and intensity of staining. Intensity was scored as 0 (no staining), 1 (weak), 2 (medium), and 3 (intensive). The extent was determined as 0 (no immunoreactive cells), 1 (1–25%), 2 (26–50%), 3 (51–75%), and 4 (> 75%). The final IHC score (ranging between 0 and 12) was calculated by multiplying the score of extent and intensity. CR-1 expression level was viewed as high when the last scores were ≥ 6 and low when the last scores were < 6. Three board-certified uropathologists with over 10 years of experience assessed the staining in a blinded manner. In the few occurrences of inconsistent scoring, a consensus score was resolved with a Multi Head Microscope. “Consensus” was defined when at least 2 reviewers reached an agreement.

### Cell lines

Human ccRCC cell lines (786-O and Caki-1) were obtained from the American Type Culture Collection (Rockville, MD). Another 3 human RCC cell lines (769-P, A498, and Caki-2) and a normal human renal tubular epithelial cell line HK-2 were obtained from the Cell Bank of the Chinese Academy of Sciences (Shanghai, China). Human umbilical vein endothelial cells (HUVECs) were obtained from ScienCell Research Laboratories (Carlsbad, CA, USA), and maintained in endothelial cell medium (ScienCell). HK-2 cells were maintained in F-12 medium (Gibco Life Technologies, Grand Island, NY), and the other cells were cultured in RPMI-1640 medium (HyClone Laboratories, Logan, UT) with 10% fetal bovine serum (FBS). All cells were cultured at 37 °C with a humidified atmosphere containing 5% CO_2_.

### ELISA assay

Quantification of serum CR-1 concentration was performed using a commercially available anti-human CR-1 ELISA kit following the instructions of manufacturer (R&D Systems, Minneapolis, MN, USA). All assays were run in duplicate at a suitable dilution, and the technicians were blinded to clinical information.

### Vector construction and lentivirus infection

To overexpress CR-1, the lentiviral vector encoding human CR-1 cDNA (LV-CR-1) was constructed by GeneChem (Shanghai, China). The empty vector was utilized as a negative control (LV-vector). To create CR-1 stable knockdown cells, the lentiviral containing CR-1 short hairpin RNA (LV-shCR-1) and the non-targeting negative control shRNA (LV-shNC) were obtained from GeneChem (Shanghai, China). The target for CR-1 were as follows: shRNA#1:5′-GCTAAATGGAAGGGCAAGTTT-3′; shRNA#2:5′-ACAGCACAGTAAGG AGCTAAA-3′; shRNA#3:5-CGCUUCUCUUACAGUGUGA-3′.In the current study, we utilized shCR-1#1 in the following experiments on the grounds that it could effectively downregulate endogenous CR-1 based on our preliminary experiments.

### MTT assay

Cells were seeded into a 96-well tissue culture plate (5 × 10^3^ per well). Then, 20 μl MTT solution (5 mg/ml) was added into each well. The cells were incubated (37 °C, 5% CO_2_) for 4 h, after which the culture media was removed, and the resultant MTT formazan was resuspended in 200 μl DMSO. The absorbance intensity at 490 nm was measured by a microplate reader (Bio-Rad).

### Colony formation assay

Approximately 1000 cells were seeded in triplicate onto a 6-well plate. After a period of 12 days, crystal violet solution (0.1%) was used to stain the cells, and the visible colonies were observed and counted manually.

### Cell cycle assay

This assay was performed with the DNA Content Quantitation Assay Kit (Solarbio, Beijing, China) according to the manufacturer’s instruction. Cells seeded in 6-well plates were harvested and fixed in 70% ethanol and stored at 4 °C overnight. After washing twice with PBS, the cells were then incubated with RNase at 37 °C for 30 min, and stained with propidium iodide for 30 min. Subsequently, the cell cycle profile was analyzed with a flow cytometry (Beckman, Fullerton, CA).

### Wound healing assay

Cells were seeded into 6-well plates and incubated until achieving full confluence as a monolayer. A wound was made by scratching the monolayer using a pipette tip across the center of the well. The cells were then washed twice gently with PBS to remove the detached cells, after which the cells were cultured with fresh serum-free media. Wound closure was photographed immediately and 24 h under a microscope.

### Migration and invasion assays

The cell migratory and invasive capability was measured utilizing transwell chambers (8 μm pore; BD Biosciences). For migration assay, cells (5 × 10^4^) were placed into the upper chamber. For invasion assay, cells (1 × 10^5^) suspended with serum-free medium were seeded into the upper chamber pre-coated with Matrigel (1:2; BD Bioscience), and the lower chamber was loaded with media containing the chemoattractant (10% FBS). After 24 h of incubation, the inserts were stained with crystal violet. The cells were carefully cleaned away from the upper chamber by a cotton tip, and the migrated or invaded cells were photographed using a microscope. Five random fields were analyzed for each chamber.

### Tube formation assay

The 96-well plates were maintained for 2 h at 4 °C and coated with 60 μl Matrigel per well. The plates were incubated for 30 min at 37 °C. Then, HUVECs (5 × 10^4^) were suspended in 100 μl indicated conditioned media, and cultured with or without anti-VEGF neutralizing antibody (ab). After incubation at 37 °C for 8 h, the tubes were observed under an inverted microscope and the total number of tube branch points were counted in 5 random fields.

### Chick embryo chorioallantoic membrane (CAM) assay

In brief, fertilized chicken eggs were incubated for 3–4 days in a humidified atmosphere at 37 °C. After this incubation, a razor and tweezers were used to create a 25-mm diameter window, and a 1% methylcellulose solution containing conditioned media was implanted inside a silicon ring which was previously fixed on the surface of CAM. After further incubation for 3 days, 2–3 ml intralipose was injected into the CAM, and the membrane was inspected under a microscope and the total number of newly growth vessels was calculated.

### Immunofluorescence (IF) staining

For IF study, cells were incubated overnight at 4  °C with primary antibodies at a dilutions of 1:200, followed by incubated with Alexa Fluor 594-conjugated secondary antibody (Invitrogen). The samples were co-stained with 4′,6-diamidino-2-phenylindole (DAPI) and observed through confocal microscopy.

### Tumorigenesis and metastasis in nude mice

Female BALB/c athymic nude mice (4–6 weeks old) were acquired from the Medical Experimental Animal Center of Guangdong province (Guangzhou, China). All mice were bred under specific pathogen-free conditions abiding by the rules of the Institutional Animal Care. To evaluate the effect of CR-1 on tumorigenic potential in vivo, the mice (5 mice /group) were injected subcutaneously with Caki-1/LV-shNC or Caki-1/LV-shCR-1 cells (5 × 10^6^ cells/mice) on the right flank. Xenograft growth was determined utilizing a caliper every 4 days. Tumor volume (V) was estimated by measurement of length (L) and width (W) of the tumor and was determined with the eq. V = (L × W^2^)/2. After 5 weeks, the mice were sacrificed, and the tumors were excised, weighed and measured. A small part of the xenograft was fixed with 4% paraformaldehyde, embedded in paraffin and subsequently stained by IHC. The remaining xenografts were rapidly placed in liquid nitrogen and used for Western blot analysis. To study the effect of CR-1 on tumor metastasis, the Caki-1/LV-shNC or Caki-1/LV-shCR-1 cells (2 × 10^6^ cells/mice) were implanted into the nude mice (5 mice /group) via tail vein injection. Six weeks later, the mice were sacrificed and the lungs were dissected and embedded in paraffin. Consecutive 4-μm sections were made and stained with haematoxylin-eosin. Lung metastatic lesions were determined by a dissecting microscope. All animal studies were approved by Gannan Medical University Animal Research Committee.

### Statistical analysis

Statistical analyses were conducted with the SPSS software package (version 19.0; IBM, Armonk, NY). The chi-square test was applied to analyze the categorical variables. The quantitative variables were analyzed utilizing the Student *t* test, Mann–Whitney U test or Kruskal–Wallis test. Pearson correlation was applied to examine the correlation between 2 quantitative variables. The receiver operating characteristic (ROC) curve was applied to determine the diagnostic potential of CR-1. Survival curves were produced utilizing the Kaplan-Meier method, and differences were estimated by the log-rank test. Univariate and multivariate analyses were performed according to the Cox proportional hazard model. In all tests, a *P*-value of less than 0.05 was viewed as statistically significant.

## Results

### Expression of CR-1 mRNA and protein in ccRCC tissues and cells

**Fig. 1:**
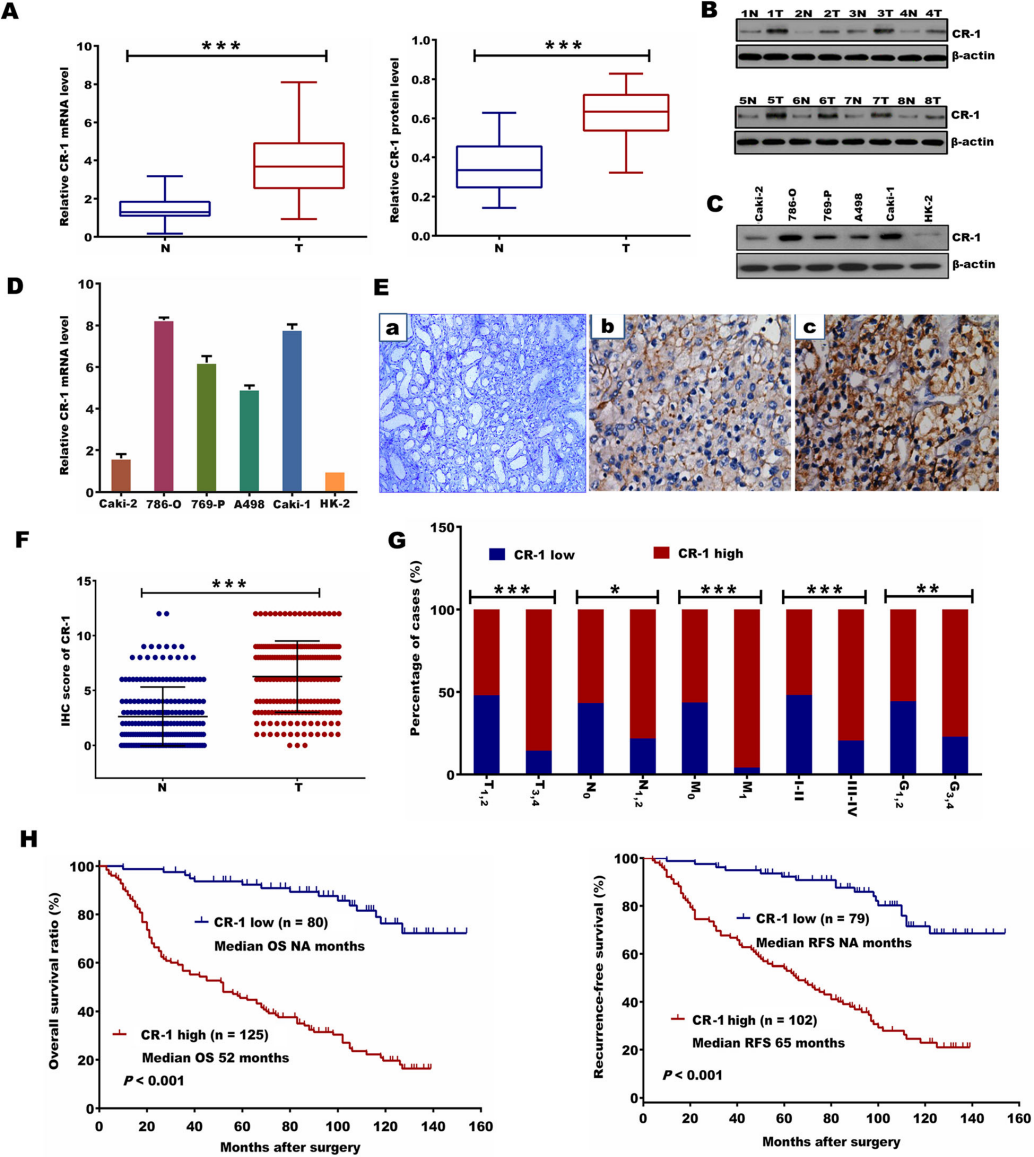
CR-1 expression is significantly up-regulated in ccRCC cells and tissues and high CR-1 expression predicts poor prognosis. (A) CR-1 mRNA and protein levels (left and right panels, respectively) in 38 paired ccRCC tumor tissues (T) and adjacent non-tumor tissues (N) were analysed by qRT-PCR and Western blot, respectively, with β-actin used as the calibrator. Data are presented as box-and-whisker plot. The line inside the boxes indicates median values with the upper and lower limits corresponding to the 75th and 25th percentiles, respectively. The upper and lower horizontal whiskers denote the 95th and 5th percentiles respectively. *P* values were calculated using a Student’s paired *t*-test, ***P <  0.001. (B) Expression of CR-1 protein in 8 representative pairs of ccRCC tissues is presented. (C) CR-1 protein expression was determined in several human ccRCC cell lines (Caki-2, 786-O, 769-P, A498, and Caki-1) and human normal renal proximal tubule epithelial cell line HK-2 by Western blot. (D) CR-1 mRNA expression was detected in the HK-2 cell line and the indicated ccRCC cell lines by qRT-PCR. Data are represented as mean ± standard deviation (SD) of three individual experiments. The relative abundance of CR-1 mRNA expression in HK-2 cells was arbitrarily designated as 1. (E) Representative IHC staining images in ccRCC and adjacent non-tumor tissues. (a) Negative CR-1 staining in adjacent non-tumor tissues. (b) Weak staining of CR-1 in ccRCC tissues. (c) Strong staining of CR-1 in ccRCC tissues. Original magnification, × 400. (F) The IHC score of CR-1 in ccRCC tissues was markedly higher than that of adjacent non-tumor tissues. Data are shown as mean ± SD. ***P <  0.001 by two-sided unpaired Student’s *t* test. (G) CR-1 protein expression was associated with T stage, lymph-node status, distant metastasis, TNM stage and Fuhrman grade. *P <  0.05, **P < 0.01, ***P < 0.001 by Chi-square test. (H) Kaplan–Meier analysis of overall survival (OS) for all patients and recurrence-free survival (RFS) for non-metastatic patients is shown based on CR-1 expression. Left and right panels indicate OS and RFS, respectively. Log-rank test was used to calculate *P* values

The CR-1 expression was first measured in 38 matched pairs of adjacent non-tumor tissue samples and fresh ccRCC samples. As compared to the matched adjacent non-tumor tissues, a statistically significant elevation of CR-1 mRNA was detected in tumors (*P* <  0.001; Fig. 1a). Similar to the mRNA results, CR-1 protein was also significantly increased in tumor tissues than in adjacent non-tumor tissues (*P* <  0.001; Fig. 1a). The protein level for CR-1 in 8 samples of representative pairs is given in Fig. 1b. The expression of CR-1 mRNA and protein was also analyzed in several RCC cell lines and an immortalized human normal proximal tubule epithelial cell line HK-2. As shown in Fig. 1c, CR-1 protein expression was higher in all 5 RCC cell lines compared to the HK-2 cell line. Also, CR-1mRNA expression was increased in those RCC cell lines relative to the HK-2 cell line (Fig. 1d).

### IHC analysis of CR-1 expression in ccRCC samples and its relationship to clinicopathological parameters

IHC was done on sections of paired adjacent non-tumor tissues and ccRCC specimens from 205 patients as well as in 8 cases of normal renal tissue. The results showed that CR-1 was primarily localized in the cytoplasm of tumor cells. High CR-1 expression was found in 125 of the 205 (60.9%) ccRCC specimens, compared with 39/205 (19.1%) in adjacent non-tumor tissues (*P* <  0.001). None of the normal renal tissues was positive for CR-1. Representative IHC images are provided in Fig. 1Ea-c. Compared to the adjacent non-tumor tissues, the IHC score of CR-1 was significantly higher in the ccRCC tissues (6.26 ± 3.25 vs 2.63 ± 2.67, *P* <  0.001; Fig. 1f). We also studied the relationship between CR-1 expression and the clinicopathological characteristics, and found that CR-1 was associated significantly with T stage (*P* <  0.001), lymph-node status (*P* = 0.013), distant metastasis (*P* <  0.001), TNM stage (*P* <  0.001) and Fuhrman grade (*P* = 0.008; Fig. 1g). No significant correlations were found between CR-1 expression and gender (*P* = 0.460) and age (*P* = 0.471). Table 1 provided the general summarization.

**Table 1:** CR-1 protein expression in 205 ccRCC tissues determined by immunohistochemistry

Variable	Patients	No. of patients (%)		*P-*value
		CR-1 Low	CR-1 High	
Age, years (median 59)				
≤ 59	107	41 (38.3)	66 (61.7)	0.471
> 59	98	39 (39.8)	59 (60.2)	
Gender				
Male	128	47 (36.7)	81 (63.3)	0.460
Female	77	33 (42.9)	44 (57.1)	
T stage				
pT_1_-pT_2_	150	72 (48.0)	78 (52.0)	< 0.001
pT_3_-pT_4_	55	8 (14.5)	47 (85.5)	
Lymph-node status				
N_0_	164	71 (43.3)	93 (56.7)	0.013
N_1–2_	41	9 (22.0)	32 (78.0)	
Distant metastasis				
M_0_	181	79 (43.6)	102 (56.4)	< 0.001
M_1_	24	1 (4.2)	23 (95.8)	
TNM stage				
I-II	137	66 (48.2)	71 (51.8)	< 0.001
III-IV	68	14 (20.6)	54 (79.4)	
Fuhrman Grade				
G_1_-G_2_	153	68 (44.4)	85(55.6)	0.008
G_3_-G_4_	52	12 (23.1)	40 (76.9)	

### CR-1 expression and patient survival

**Fig. 2:**
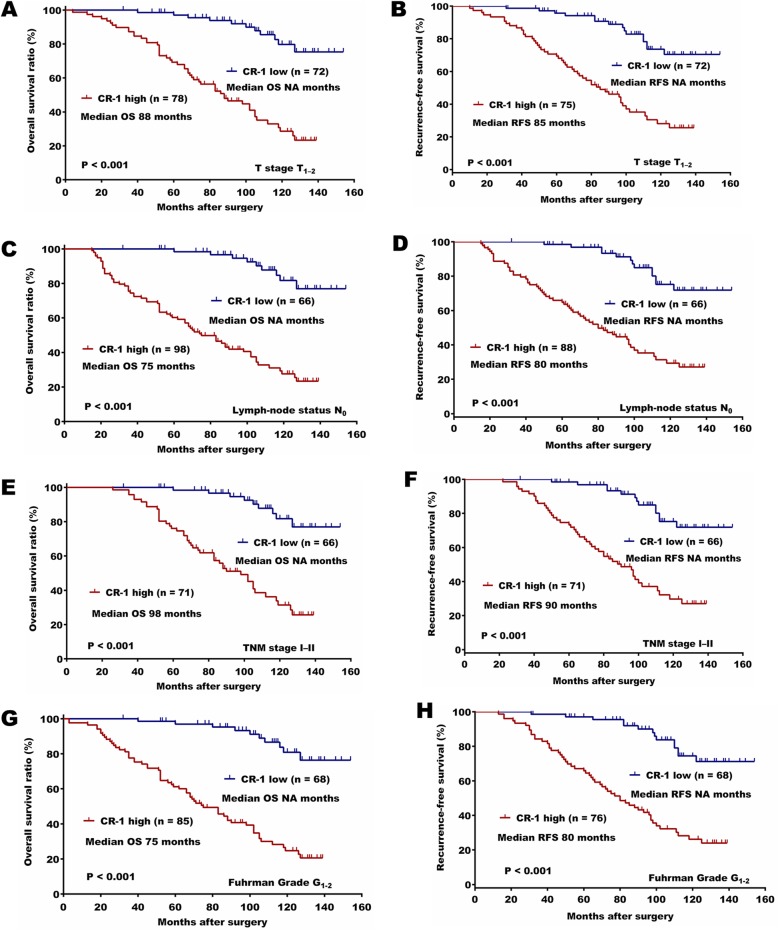
Kaplan-Meier survival analysis for CR-1 is shown in other ccRCC subgroups. **a** and **b** Prognostic role of CR-1 in patients with T stage T_1–2_, **c** and **d** with lymph node status N_0_,. **e** and **f** with TNM stage I-II, and **g** and **h** with Fuhrman grade G_1–2_. Log-rank test was used to calculate *P* values

The Kaplan-Meier survival analysis showed that patients with high expression of CR-1 had significantly shorter OS and RFS than those with low expression of CR-1 (both *P* <  0.001; Fig. 1h). This results were further confirmed by the univariate analysis, in which high expression of CR-1 was significantly related to poor patient survival (OS, HR, 7.031, 95% CI, 4.068–12.152, *P* <  0.001; RFS, HR, 5.133, 95% CI, 3.058–8.616, *P* <  0.001; Table 2A and B). Furthermore, multivariate analysis revealed that high expression of CR-1 was an independent predictor for both OS and RFS (OS, HR, 6.518, 95% CI, 3.689–11.516, *P* <  0.001; RFS, HR, 5.638, 95% CI, 3.274–9.711, *P* <  0.001). Predictive value of CR-1 in the low stage or low grade subgroup of ccRCC, such as in those with T stage T_1–2_, Fuhrman grade G_1–2_, lymph node status N_0_, and TNM stage I-II subgroups were studied further. The prognostic significance of CR-1 was also observed in those subgroups (Fig. 2a-h).

**Table 2:** Cox regression analysis for overall survival and recurrence-free survival

Variable	Univariate analysis			Multivariate analysis		
	HR	95%CI	*P*	HR	95%CI	*P*
(A) Overall survival						
T stage						
T_3–4_ vs T_1–2_	7.417	4.985–11.035	< 0.001	2.531	1.547–4.141	< 0.001
Lymph-node status						
N_1–2_ vs N_0_	7.141	4.681–10.895	< 0.001	3.969	2.431–6.482	< 0.001
Distant metastasis						
M_1_ vs M_0_	13.621	7.747–23.946	< 0.001	3.809	2.074–6.995	< 0.001
Fuhrman grade						
G_3–4_ vs G_1–2_	4.594	3.067–6.883	< 0.001	1.946	1.198–3.161	0.007
CR-1 expression						
High vs low	7.031	4.068–12.152	< 0.001	6.518	3.689–11.516	< 0.001
(B) Recurrence-free survival						
T stage						
T_3–4_ vs T_1–2_	5.380	3.409–8.490	< 0.001	2.051	1.167–3.606	0.013
Lymph-node status						
N_1–2_ vs N_0_	6.320	3.836–10.410	< 0.001	4.818	2.581–8.995	< 0.001
Fuhrman grade						
G_3–4_ vs G_1–2_	3.685	2.302–5.897	< 0.001	1.783	1.045–3.043	0.034
CR-1 expression						
High vs low	5.133	3.058–8.616	< 0.001	5.638	3.274–9.711	< 0.001

Abbreviations: *HR* Hazard ratio, *95% CI* 95% confidence interval

### Serum CR-1 expression assessed by ELISA

**Fig. 3:**
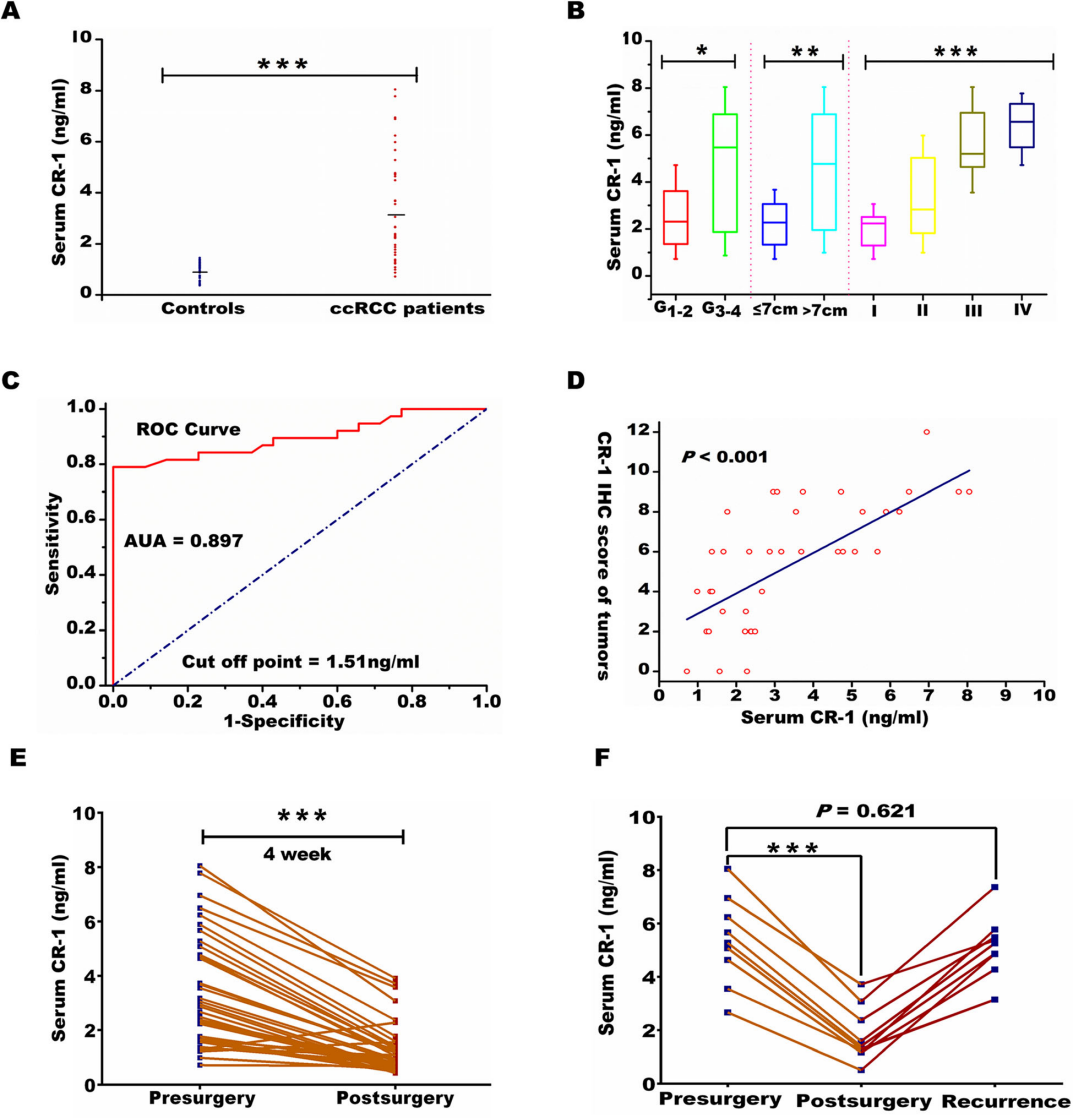
Serum level of CR-1 in ccRCC patients. **a** Comparison of serum CR-1 levels in healthy controls (*n* = 35) and ccRCC patients (*n* = 38) using ELISA. Horizontal bars represent the mean. *P* value was calculated using the Mann–Whitney U-test, ***P < 0.001. **b** comparison of CR-1 concentration stratified by Fuhrman grade (≤2 vs  > 2), tumor size (≤7 vs  > 7 cm), and TNM stage (I-IV). *P < 0.05, **P < 0.01 by Mann–Whitney U-test; ***P < 0.001 by Kruskal–Wallis test. **c** Receiver operating characteristic (ROC) curve analysis of serum CR-1 for distinguishing patients with ccRCC from healthy controls. **d** Comparison between CR-1 serum concentrations and CR-1 expression in ccRCCs. The CR-1 expression levels are presented as IHC scores. Linear correlation is examined using Pearson’s correlation analysis (Pearson coefficient 0.735, *P* < 0.001). **e** At the fourth week after surgery, the serum CR-1 levels were remarkably decreased compared with their preoperative serum. ***P < 0.001 by Student’s paired *t*-test. **f** In 10 patients with documented recurrence, the serum CR-1 were increased again to the preoperative levels. ***P < 0.001 by Student’s paired *t*-test. *P* = 0.621 by Mann–Whitney U-test

Further, The CR-1 serum levels were determined, and CR-1 concentrations were notably higher in ccRCC patients (*n* = 38) than in healthy controls (*n* = 35) (3.34 ± 2.08 ng/mL vs 1.04 ± 0.33 ng/ml, *P* <  0.001; Fig. 3a). CR-1 values were significantly correlated with Fuhrman grade (≤2 vs  > 2; *P*   =   0.015), tumor size (≤7 vs  > 7 cm; *P*   =  0.005), and TNM stage (*P <* 0.001; Fig. 3b). ROC analysis led to an optimal cutoff value of 1.51 ng/ml for serum CR-1 level, which had the highest area under the ROC curve (area = 0.897; sensitivity = 78.9%; specificity = 100%; Fig. 3c). Moreover, high CR-1 serum levels were markedly correlated to high CR-1 expression in tumor tissues as evaluated by IHC (Pearson correlation coefficient = 0.735, *P* <  0.001; Fig. 3d). The dynamic alterations of postoperative CR-1 serum levels were monitored. The CR-1 serum levels were remarkably decreased compared to their preoperative serum by the 4th week after operation (*P* <  0.001; Fig. 3e), suggesting that CR-1 could reflect tumor burden. Moreover, in 10 cases with documented recurrence, the decreased serum CR-1 levels after surgery were increased again at the time of tumor recurrence (*P* = 0.621; Fig. 3f).

### CR-1 promotes ccRCC cell proliferation and tumorigenicity in vitro and in vivo

**Fig. 4:**
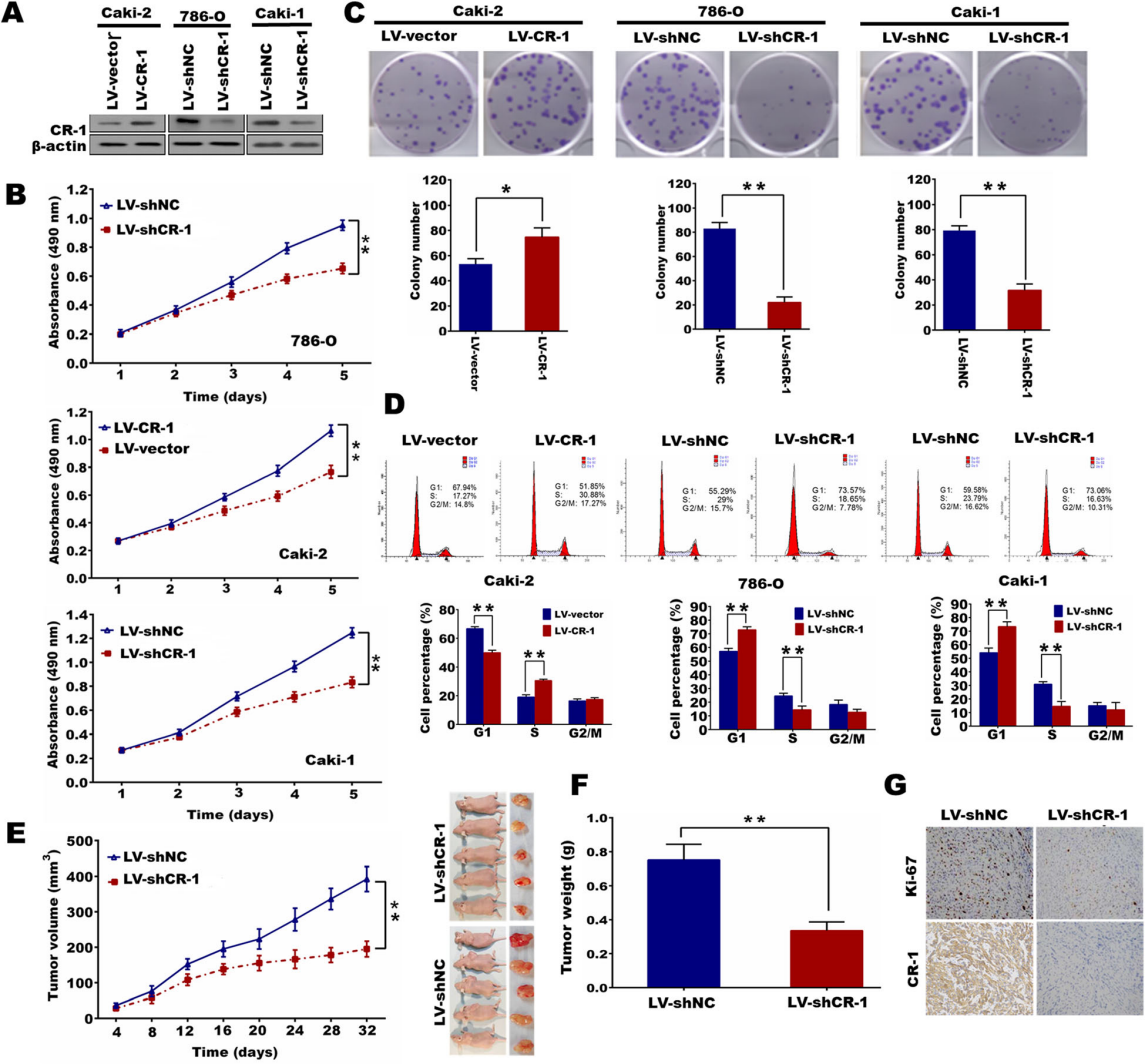
CR-1 promotes ccRCC cell proliferation and tumorigenicity in vitro and in vivo*.* **a** CR-1 protein is increased after over-expression of CR-1 in Caki-2 cells and decreased after knockdown of CR-1 in 786-O and Caki-1 cells. **b** and **c** Ectopic expression of CR-1 stimulates cell proliferation in Caki-2 cells whereas knockdown of CR-1 inhibits cell proliferation in 786-O and Caki-1 cells as determined by MTT assays (**b**) and colony formation assays (**c**). Data represent the mean ± SD of three independent experiments. *P < 0.05, **P < 0.01 by two-sided unpaired Student’s *t*-test. **d** Cell cycle analysis was performed by flow cytometry for indicated cell lines. The percentage of cell population at different cell cycle phases are shown in the histograms. **P < 0.01 by two-sided unpaired Student’s *t*-test. **e** Effects of CR-1 knockdown on tumor growth in vivo. Caki-1/LV-shNC or Caki-1/LV-shCR-1 cells (5 × 10^6^) were injected subcutaneously into nude mice (5 mice /group). Left panel, tumor growth curves. Right panel, images of tumor xenografts. Data are represented as mean ± SD. **P < 0.01 by two-sided unpaired Student’s *t*-test. **f** Comparison of weight of harvested xenografts. Data are presented as means ± SD. **P < 0.01 by two-sided unpaired Student’s *t*-test. **g** Histopathology of xenograft tumors, the tumor sections were under IHC staining for CR-1 and Ki-67

In light of the information listed above, the role of CR-1 in cell proliferation was further assessed. The Caki-2 cell line, which owned relatively low CR-1 expression, was infected with LV- CR-1 to overexpress CR-1. Another two cell lines 786-O and Caki-1 that expressed relatively high level of CR-1 were dealt with LV-shCR-1 to knockdown CR-1 endogenous expression. The efficacy of ectopic expression or knockdown of CR-1 in cells was verified by Western blot (Fig. 4a). MTT assay demonstrated that CR-1 ectopic expression could significantly facilitate the proliferative capacity in Caki-2 cells as compared to control cells (*P* <  0.01; Fig. 4b). Likewise, colony formation capacity was markedly increased after overexpression of CR-1 (*P* <  0.01, Fig. 4c). In contrast, knockdown of CR-1 impeded the growth ability of 786-O and Caki-1 cells as indicated by the MTT and colony formation assays (*P* <  0.01; Fig. 4b and c). To understand the mechanism by which CR-1 enhanced ccRCC cell proliferation, we performed flow cytometry to measure the cell cycle distribution.

Our data showed that 786-O and Caki-1 cells with CR-1 knockdown exhibited a significant accumulation in G1 phase and a remarkable decrease in S phases as compared with those in the matched controls, whereas CR-1 overexpression significantly decreased the percentage of Caki-2 cells in G1 phase and increased that in S phase (*P* <  0.01; Fig. 4d). To analyze whether CR-1 shRNA had the effect on tumor growth inhibition in vivo, a nude mice xenograft model was established from Caki-1 cells. The results demonstrated that tumor growth in shRNA group was significantly suppressed compared to control group (*P* <  0.01; Fig. 4e). Moreover, CR-1 shRNA led to a significant reduction of the tumor weight as assessed at the completion of the experiment when compared to control group (*P* <  0.01; Fig. 4f). Apart from the difference in tumor volume and weight, we also found the control group exhibited much stronger CR-1 and Ki-67 staining, as detected by IHC (Fig. 4g).

### CR-1 promotes ccRCC cell migration, invasion and angiogenesis in vitro and in vivo

**Fig. 5:**
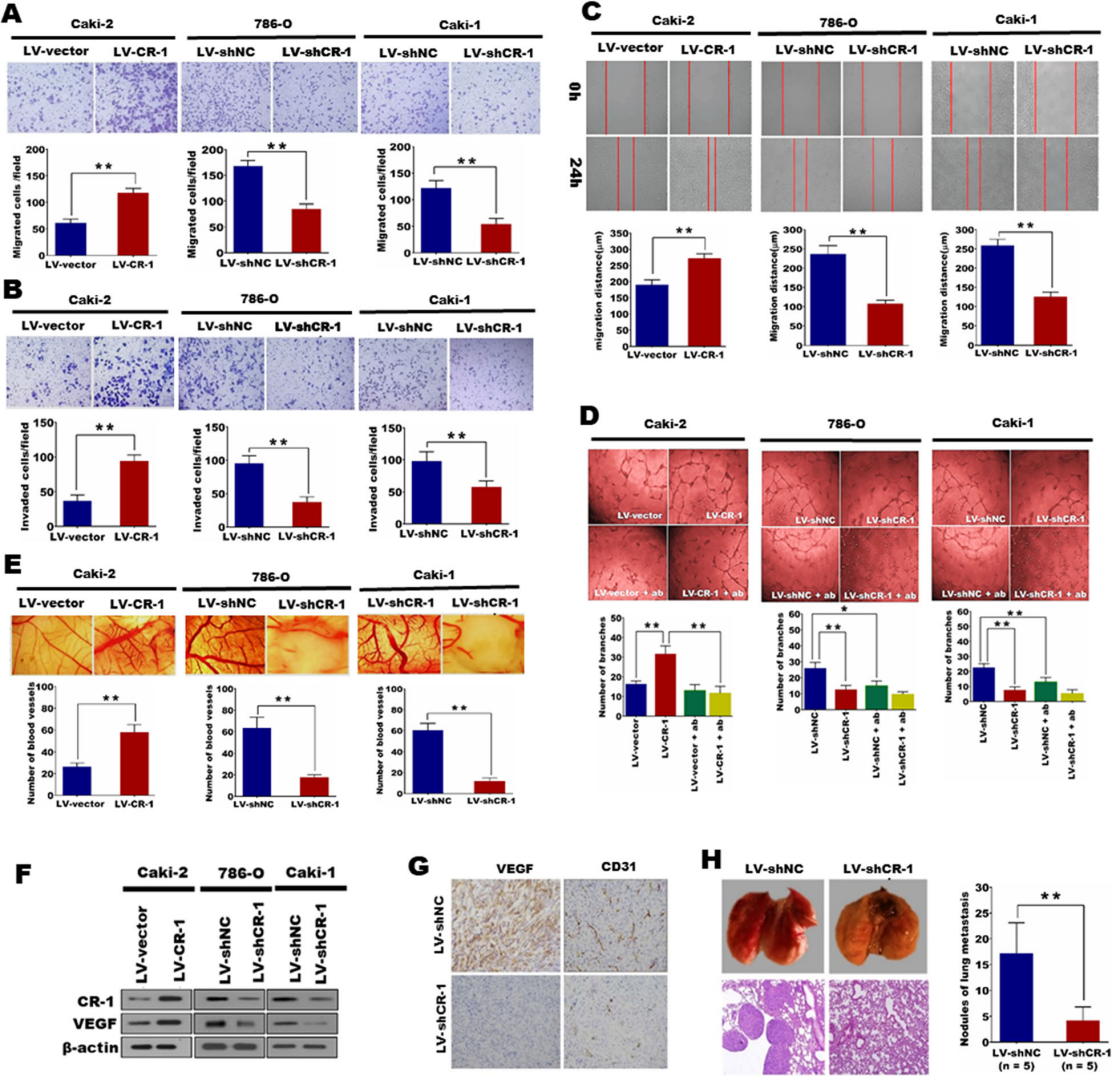
CR-1 promotes ccRCC cell migration, invasion and angiogenesis in vitro and in vivo. **a** and **b** Ectopic expression of CR-1 stimulates cell migration and invasion in Caki-2 cells whereas knockdown of CR-1 inhibits cell migration and invasion in 786-O and Caki-1 cells as determined by Transwell migration (**a**) and Matrigel invasion assays (**b**). Data represent the mean ± SD of three independent experiments. **P < 0.01 by two-sided unpaired Student’s *t*-test. **c** Ectopic expression of CR-1 enhances cell migration whereas knockdown of CR-1 inhibits cell migration as determined by the wound healing assay. Photomicrographs were obtained at 0 and 24 h and migrated distance was measured using Image J software (Bethesda, USA). Data represent the mean ± SD of three independent experiments. **P < 0.01 by two-sided unpaired Student’s *t*-test. **d** Tube formation assay was performed with HUVECs cells incubated in culture supernatant fluid of indicated cell lines in the presence or absence of anti-VEGF neutralizing antibody. Data represent the mean ± SD of three independent experiments. *P < 0.05, **P < 0.01 by two-sided unpaired Student’s *t*-test. **e** The new blood vessel formation in CAM was quantified. Data represent the mean ± SD of three independent experiments. **P < 0.01 by two-sided unpaired Student’s *t*-test. **f** Relative protein expression of CR-1 and VEGF as determined by Western blot for indicated cell lines. **g** IHC staining for CD31 and VEGF in tumor specimens from xenografts. Original magnification, × 100. **h** In vivo metastasis assay was performed in nude mice (5 mice /group) injected with Caki-1/LV-shNC or Caki-1/LV-shCR-1 cells (2 × 10^6^). Left panel, representative images and HE staining of lungs. Original magnification, × 200. Right panel, numbers of tumor nodules in each group. Data represent mean ± SD. **P < 0.01 by two-sided unpaired Student’s *t*-test

Cell migration and invasion were measured using Transwell assays. As compared with control cells, the migration and invasion capacity were distinctly enhanced in Caki-2 cells which overexpressing CR-1 (*P* <  0.01; Fig. 5a and b). Conversely, knockdown of CR-1 could restrict the migration and invasion of 786-O and caki-1 cells apparently (*P* <  0.01; Fig. 5a and b). Wound healing assay was further applied to assess the effect of CR-1 on cell migration. The results demonstrated that CR-1 overexpression enhanced migration ability in Caki-2 cells (*P* <  0.01; Fig. 5c), whereas CR-1 knockdown inhibited the migration of 786-O and Caki-1 cells (*P* <  0.01; Fig. 5c). Next, we studied the in vitro effect of CR-1 on tumor angiogenesis by HUVECs tube formation assay. Our results demonstrated that the tube formation was increased significantly when the HUVECs were cultured in conditioned media from Caki-2 cells infected with LV-CR-1 compared to the control group cells (*P* <  0.01; Fig. 5d). However, the Caki-1 and 786-O cells infected with LV-shCR-1 exhibited opposite effect (*P* <  0.01; Fig. 5d). Besides, when VEGF neutralizing antibody was used to neutralize VEGF in the culture supernatants of HUVEC cells, the tube formation of the cell induced by CR-1 were markedly abolished in vitro (*P* <  0.01; Fig. 5d). Additionally, we further investigate the efficacy of CR-1 on angiogenesis using the CAM assay, and found that overexpression of CR-1 could significantly improve the new blood vessels formation in Caki-2 cells (*P* <  0.01; Fig. 5e) while knockdown of CR-1 could reduce the blood vessels formation in Caki-1 and 786-O cells (*P* <  0.01; Fig. 5e). Moreover, we found that the expression of VEGF was significantly decreased when knocking down CR-1 in Caki-1 and 786-O cells (Fig. 5f), whereas it was dramatically increased in Caki-2 cells that overexpressing CR-1 (Fig. 5f). We also detected noticeably reduced VEGF protein expression in Caki-1 cells xenografts with knockdown of CR-1 by IHC (Fig. 5g). In addition, the CD31-postive microvascular was greatly decreased with the knockdown of CR-1 in the xenograft tissues (Fig. 5g). We further evaluated whether knockdown of CR-1 repressed the metastasis in vivo. Caki-1/LV-shCR-1 and Caki-1/LV-shNC cells were injected into nude mice via tail vein. Six weeks after injection, mice were sacrificed and lung metastatic burden were assessed. The frequency of lung metastases was significantly lower (*P* <  0.01; Fig. 5h) in nude mice injected with LV-shCR-1 cells compared to LV-shNC cells.

### CR-1 promotes EMT in ccRCC cells by activating the Wnt/β-catenin signaling in vitro and in vivo

**Fig. 6:**
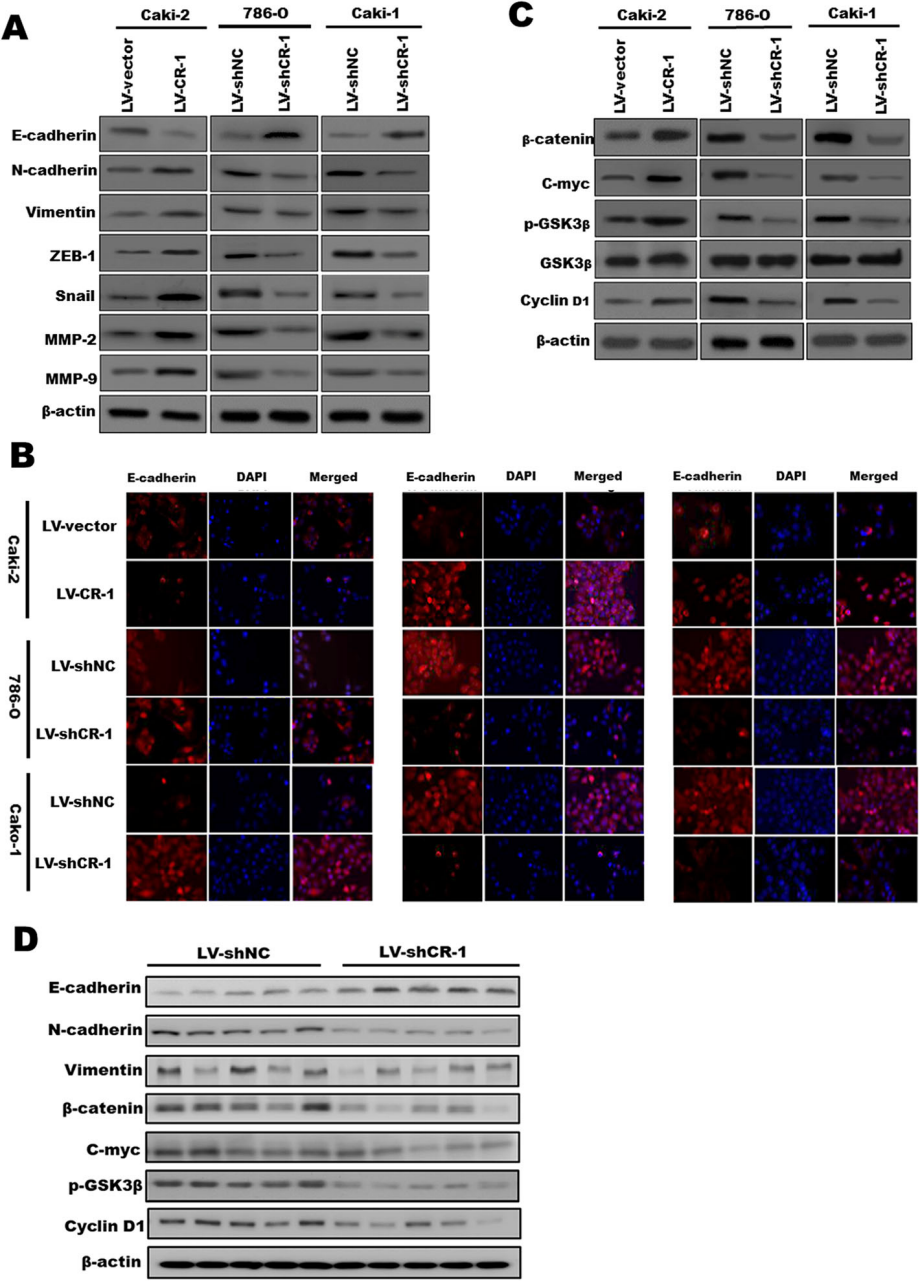
CR-1 promotes EMT in ccRCC cells by activating the Wnt/β-catenin signaling in vitro and in vivo. **a** After overexpressing CR-1 in Caki-2 cells or downregulating CR-1 expression in 786-O and Caki-2 cells, the protein levels of E-cadherin, N-cadherin, Vimentin, ZEB-1, Snail, MMP-2, and MMP-9 were measured by Western blot. **b** IF was used to compare the expression levels of E-cadherin, N-cadherin, and Vimentin between Caki-2/LV- vector and Caki-2/LV- CR-1, 786-O/LV-shNC and 786-O/LV-shCR-1, and Caki-1/LV- shNC and Caki-1/LV-shCR-1 cells. **c** CR-1 knockdown reduced the expression of p-GSK3β, β-catenin, C-myc, and cyclin D1; in contrast, CR-1 upregulation increased the expression of p-GSK3β, β-catenin, C-myc, and cyclin D1. **d** Expression of EMT markers (E-cadherin, N-cadherin and Vimentin,) and Wnt/β-catenin signaling genes (β-catenin, p-GSK3β, C-myc, and cyclin D1) was detected by Western blot using xenograft tumors from Caki-1/LV- shNC (*n* = 5) and Caki-1/LV-shCR-1 (n = 5) groups

Because EMT is critical for the acquisition of invasive and metastatic properties in tumors, we measured EMT markers by Western blot to evaluate whether CR-1 could promote EMT-mediated ccRCC invasion. We found that the expression of Vimentin, N-cadherin, ZEB-1, and Snail was increased, while the expression of E-cadherin was decreased, after overexpressing CR-1 in Caki-2 cells, and the opposite effects were acquired when CR-1 was knocked down in Caki-1 and 786-O cells (Fig. 6a). Additionally, we studied the expression of matrix metalloproteinases (MMPs), which stimulate tumor invasion and metastasis via the degradation of extracellular matrix, and found that CR-1 overexpression up-regulated MMP-2 and MMP9 expression, conversely, silencing of CR-1 expression exhibited opposite results (Fig. 6a). Moreover, IF analysis was carried out to analyze the protein expression of E-cadherin, N-cadherin, and Vimentin in ccRCC cell lines (Fig. 6b), and these results were in line with those of the Western blot assays. As Wnt/β-catenin pathway plays a crucial role in induction and maintenance of EMT, we detected the protein expression of the Wnt/β-catenin signaling genes for β-catenin, p-GSK3β, C-myc, and cyclin D1 in Caki-2 cells overexpressing CR-1 or in which CR-1 had been knocked down in 786-O and Caki-1 to explore the CR-1 mechanism underlying ccRCC metastasis. The results indicated that CR-1 knockdown decreased the expression of β-catenin, p-GSK3β, C-myc, and cyclin D1; conversely, CR-1 overexpression increased the expression of β-catenin, p-GSK3β, C-myc, and cyclin D1 (Fig. 6c). Furthermore, we assessed the effects of CR-1 on the EMT and Wnt/β-Catenin signaling pathway in vivo*.* In line with the in vitro findings, Western blot of the β-catenin, p-GSK3β, C-myc, Cyclin D1 and EMT markers showed the same changing trend in xenograft tissues from Caki-1 cells infected with LV-shCR-1 or LV-shNC (Fig. 6d). These results showed that CR-1 could facilitate ccRCC cell migration and invasion by induction of EMT and activation of Wnt/β-Catenin signaling.

## Discussion

Accumulating evidence has demonstrated that high expression of CR-1 might be a key alteration contributing to the invasion and metastasis of tumor cells [11, 13, 31]. Recently, CR-1 was also found to be associated with prognosis in several kinds of tumors [14–21]. Until now, systematic investigation of the prognostic significance and biological role of CR-1 in ccRCC has not been reported, especially with long-term follow-up and a large number of patients. Thus, the role of CR-1 in ccRCC progression has not been clearly defined.

This investigation made several discoveries concerning the role of CR-1 in the malignant progression of ccRCC. First, IHC analysis of clinical samples indicated that there was a positive correlation between CR-1 expression and aggressive tumor phenotype and poor survival. Second, serum CR-1 was significantly elevated in most ccRCCs, which may serve as a novel marker and postoperative monitoring of ccRCCs. Third, we demonstrated that CR-1 facilitated ccRCC cell proliferation, migration, invasion, and angiogenesis as well as tumorigenesis and metastasis both in vitro and in vivo. Finally, we showed that CR-1 induced EMT and activated Wnt/β-catenin signaling pathway. These data suggest that CR-1 has an important role in ccRCC progression. CR-1 protein could be an attractive anticancer target.

The present study immunohistochemically analyzed 205 tumor samples of ccRCC. The results indicated that high CR-1 expression was 125/205 (60.9%) in ccRCC tissues compared with 39/205 (19.1%) in adjacent non-tumor tissues (*P* <  0.001). Previous studies have shown that CR-1 expression is present in embryonic tissues and becomes silenced in postnatal tissues. Reactivation of CR-1 in adult tissues has been associated with various cancer types. However, high CR-1 expression was also observed in 39 adjacent non-tumor tissues in our study. This phenomenon may be a representation of the so called “field cancerization” of theory, suggesting a cumulative process of carcinogenesis in which genetic alterations are acquired step-wise, leaving the adjacent non-tumor tissue in an intermediate, pre-neoplastic state composed of morphologically normal but molecularly altered cells [32]. However, we should not rule out the possibility that while adjacent tissues were identified as “normal” by certified pathologists, there is a chance that a few ccRCC cells might have been missed during the exam.

In our IHC analysis, high CR-1 expression is associated significantly with aggressive tumor phenotype, which suggest that CR-1 expression may be vital for the acquisition of malignant potential in ccRCCs. In agreement with our findings, several previous IHC studies of CR-1 have also revealed the relationship between CR-1 expression and clinicopathologic features on other cancers. In gastric cancers, CR-1 expression was positively associated with lymph node metastasis, liver metastasis, and TNM stage [14]. In non-small cell lung cancer, CR-1 expression was correlated significantly with poor tumor differentiation, TNM stage, and lymph node metastasis [19]. In esophageal squamous cell carcinoma, CR-1 expression was associated significantly with depth of invasion, TNM stage and lymph node metastasis [21].

Cancer is a heterogeneous disease, and patients at the same clinical stage of disease, with similar histopathological tumor features, and similar treatment strategies (such as surgical resection) can have different clinical outcomes. Our IHC results suggest CR-1 as a novel independent marker of ccRCC outcome. Further analysis of the prognostic significance of CR-1 in clinical subgroups indicated that the OS and RFS of CR-1 high expression patients who had TNM stage I or II were dramatically worse than CR-1 low expression patients in the same stage. This suggests that CR-1 could serve as a promising predictive marker in early-stage ccRCC patients. The prescient estimation of CR-1 in this subgroup can assist clinicians to identify patients at high risk of recurrence and empower clinicians to afford reasonable adjuvant treatment in a timely fashion.

CR-1 has been established as a novel biomarker in colon, breast or cerebral tumors due to its significant presence in the plasma of such cancer patients as compared to the normal volunteers [25, 33]. In both studies, tumor tissues and patient-matched blood samples have been analyzed, showing that high CR-1 levels in the plasma correspond to re-expression of CR-1 in tumor tissues [25]. In our current study, radical nephrectomy led to a substantial reduction in serum CR-1 to a lower level, and the decreased serum CR-1 was increased again on the occasion of tumor recurrence. This results provide initial evidence of a connection between serum CR-1 levels and ccRCC recurrence that deserves further investigation. Our results also imply that the IHC examination of CR-1 protein expression in ccRCC tissue is accord well with CR-1 serum protein levels of patients as evaluated by ELISA. Accordingly, the ELISA-based method for measuring CR-1 serum concentration may yet be a more feasible approach to obtain prognostic data. However, the sample numbers of the current study are limited, and, therefore, the statistical significance found in this study may not be stable. Therefore, the credibility of serum CR-1 level as an independent prognostic marker needs to be confirmed in a larger study.

Moreover, many data indicate CR-1 as a promising target for cancer therapy. CR-1 inhibition by different approaches always resulted in inhibition of cancer cell proliferation in vitro and of tumor growth in vivo [13, 21, 22, 28]. CR-1 is believed to induce cell proliferation through multiple pathways, including ERK 1/2 activation, TGF-β/smad-2 signaling blockade, and TGF-β/activin B signaling blockade [34]. In the present study, we demonstrated that CR-1 knockdown cells were arrested in the G1 phase and thus inhibited ccRCC cell proliferation. The result was consistent with the study in prostate cancer [35]. However, a previous study by Wu et al. has suggested that CR-1 knockdown does not influence the cell cycle in nasopharyngeal carcinoma cells [36]. Possible explanation for this difference was different tumor origins. Therefore, further investigation is needed to explore the specific mechanism.

EMT has been viewed as a crucial step in tumor invasion and metastasis [37]. Decreased E-cadherin expression is regarded as a profound event in EMT [38]. Among the EMT-related transcription factors, Snail represses E-cadherin transcription through binding to E-cadherin promoter, whereas factors such as ZEB-1 repress E-cadherin indirectly. In the present study, we examined the levels of EMT markers, including E-cadherin, N-cadherin, Vimentin, Snail, and ZEB-1. The expression levels of Snail and ZEB-1 were repressed significantly after CR-1 knockdown in 786-O and Caki-1 cells, while the epithelial marker E-cadherin was upregulated. On the opposite, CR-1 overexpression enhanced EMT, showing that CR-1 plays a vital role in EMT in ccRCC. These results suggest that CR-1 knockdown repressed tumor invasion and metastasis through restraining the EMT process in ccRCC cells.

Angiogenesis is a fundamental event that governs the progression and development of malignant tumors [39]. As a pivotal regulator of angiogenesis, VEGF has been shown to play important roles not only for endothelial cell proliferation and migration but also for tumor cell survival and proliferation in an autocrine/paracrine manner [40, 41]. The functions of CR-1 in stimulating proliferation and survival of endothelial cells have been proven in a breast cancer model [42]. This implies the potential role of CR-1 as a main modulator of tumor blood vessel formation. In the current study, we found that CR-1 knockdown suppressed microtubule assembly in vitro and decreased microvascular density in vivo. Moreover, neutralizing VEGF could block CR-1 overexpressed culture medium stimulated angiogenesis in tube formation assay, which suggested an indirect effect of CR-1 on angiogenesis. These results implied that CR-1 knockdown attenuated ccRCC angiogenesis.

Multiple signaling pathways, including the NF-kB, Wnt, Notch, and TGF-β signaling pathways, are involved in regulation of EMT [43–46]. Wnt/β-catenin signaling plays a key role in EMT induction and maintenance [47, 48]. Dysregulation of Wnt/β-catenin signaling pathway is believed to enhance the malignancy in various types of human cancers, including ccRCC [49–53]. In this study, we found that CR-1 upregulation enhanced the expression of several key genes in the Wnt/β-catenin pathway; in contrast, CR-1 knockdown reduced these gene expressions. These data suggested that CR-1 promotes the EMT in ccRCC by activating the Wnt/β-catenin signaling pathway.

Several limitations to our present study merit discussion. First, our study was a single hospital-based and retrospective study. Future studies based on a multi-center or community-based prospective study are needed to confirm our results. Second, we recognize that the overall number of patient serum samples that we studied is relatively small. Larger studies are clearly warranted and may help to elucidate important potential cut points to translate CR-1 into the clinical arena as a serum tumor marker. Third, whereas this study revealed that CR-1 activated the EMT and Wnt/β-Catenin signaling pathway, it remains unproven how CR-1 regulate the expression of N-cadherin, β-Catenin, MMPs, etc. Future work should be further explored. In addition, while CR-1 has multiple signaling mechanisms that may contribute to tumor progression, its known cell surface binding partners do not appear to fully explain its reported oncogenic functions. Therefore, identification of novel CR-1 interacting proteins are needed.

## Conclusions

In conclusion, we have for the first time provided evidence that ccRCC patients displayed upregulation of CR-1 in tumor tissues and increased serum CR-1 levels. The increased expression of CR-1 in ccRCC positively correlates with the aggressive phenotype, and predicts poor clinical outcome. We have also provided clear experimental evidence that CR-1 facilitates ccRCC cell proliferation, migration, invasion, and angiogenesis as well as tumorigenesis and metastasis. More importantly, we have also provided insight into the potential molecular mechanism, and obtained evidence that CR-1 regulates the EMT and Wnt/β-catenin signaling involved in invasion and metastasis. Therefore, CR-1 expression level could be used to predict cancer progression, metastasis and prognosis of ccRCC patients. Targeting CR-1 might be a promising therapeutic strategy for ccRCC patients. Further studies clarifying the detailed mechanisms underlying the role of CR-1 in ccRCC are very interesting and are an area of active research at our institute.

## Data Availability

All data generated or analyzed during this study are included in this published article.

## Abbreviations

CAM: Chick embryo chorioallantoic membrane
ccRCC: Clear cell renal cell carcinoma
ELISA: Enzyme-linked immunosorbent assays
EMT: Epithelial-to-mesenchymal transition
IF: Immunofluorescence
IHC: Immunohistochemistry
MMPs: Matrix metalloproteinases
OS: Overall survival
qRT-PCR: Real-time quantitative PCR
RCC: Renal cell carcinoma
RFS: Recurrence-free survival
ROC: Receiver operating characteristic
VEGF: Vascular endothelial growth factor
